# Lectures on the Theory and Practice of Physic

**Published:** 1840

**Authors:** 


### REVIEWS.

---

Art. XIII.—*Lectures on the Theory and Practice of Physic.* By the late David Hosack, M. D., L. L. D., &c., Philadelphia, 1838.

---

(*Concluded from the January number.*)

---

We promised to the readers of the Louisville Journal, another notice of this production. And we are sorry we did so; because we have nothing in relation to it very palatable to offer them—nothing, we mean, of that commendation, which we would feel both proud and pleased to bestow, on an American publication in medicine, whose author had been a practitioner of his profession near half a century, and a public teacher of it a large portion of that time. And our regret on the subject is heightened not a little by the fact (which we deem a singular one) that the work has received a warm and studied eulogy, from the pen of another physician, who justly ranks, as a teacher and practitioner, among the most distinguished our country has produced. But no matter for all this. Our promise is out, and must be redeemed. And, as far as our time and resources may avail, it shall be redeemed faithfully.

The more we look into the "Lectures," the less are we satisfied with them; because in neither matter, manner, nor thought, do we find in them scarce a tittle in harmony with the more substantial and accurate medical science and literature of the day. Wherever we open the book, and on whatever page we fix our eyes, we find it covered and crammed, to a most offensive degree of bloatedness, by a pompous citation of authorities, and a vain-glorious display of quotations, as if the writer had been framed to walk only on *stilts*, or on the shoulders of other men. And the evil is greatly increased by the condemnable fact, that almost all the quotations, *certainly* a large majority of them, are in the Latin tongue, and were not, we venture to say, understood by one in twenty—most probably not by one in fifty, of those for whose edification they were intended. As respects the communication of instruction to them (the true and only end of didactic lectures) the learned Professor might as well have addressed most of his class in the language of Zoroaster, as in that of Cicero.

A leading object, and as we conceive a very important one, which we have in view, in our notice of the work before us, being to show, that it is a vitiated, and erroneous, and therefore a dangerous and discreditable standard for medical literature, science, and practice, at the present period, when fact and sound reason are fast ascending the high places that were formerly held by theories and notions—and farther, to show, that the true standard of *western medicine* can be erected only by *western physicians*—such being the case, and it being true, that any one portion of the work furnished us with matter nearly as well suited to our purpose as any other, we have taken but not selected, as the subject of a few remarks, "Lecture XIV," headed:

"Causes of Fever—Miasma and Contagion."

Now so trite and common-place have these topics been rendered, by the extensive and multiplied discussions they have undergone, that it would seem easy for a public teacher, when addressing his class, to concentrate on them, in a few sentences or paragraphs at the farthest, all the positive knowledge of them he possesses, and to derive ample illustrations of his opinions respecting them, from annual occurrences in our own country, and from a few of the able and accurate writers of modern times. Such however do not appear to have been the views of our learned author. To appear under the plain and sober sun-light of truth, common-sense, and practical medicine, did not suit the gorgeousness of his taste. He could content himself with nothing less than a career among the dusky and conflicting clouds of professional authorities, learning, and majesty.

Accordingly he begins with certain views on the insalubriousness of swamps and stagnant waters, entertained when medicine was young by "Hippocrates, Galen, Varro, Columella, Palladius, Vitruvius, Diodorus Siculus, Dionysius Halicarnassensis, Strabo, and others," (but what others he does not tell us, and know, nor care,) and then quotes from Sydenham a long passage in Latin to show that he knew nothing at all of the matter.

Having thus done due homage to ten or twelve ancients, most of whom, were they now alive, and possessed of no more knowledge than belonged to them when they wrote their books, would be *medical novices;* and having fastened on the great British Hippocrates the charge of being utterly ignorant of a subject, of which he himself was so thoroughly informed—having achieved these literary and scientific feats of knight errantry—he next pays a visit to Lancisci the Italian, nobody very well knows why, unless it be for the sake of telling the story, told five hundred times before, about "thirty ladies and gentlemen of Rome," of whom twenty-nine contracted a malarious fever, in consequence of a water-excursion on the Tiber. His interview with Lancisci, he closes by informing him that his able work on marsh miasma, had been accurately translated by no less a personage than Lieutenant Governor Colden, of New York, a fact known before to every reader of American medical literature.

But our author's chase after "miasma and contagion" is not yet ended. Far from it. We accordingly find him next in Asia, counting the cadavers of the Turks, who died in Bussorah, of "miasma," or "contagion" (he does not tell us which) in consequence of the stagnant water thrown around the city, by the superflux of the Euphrates. And he discovers that not less than twelve or fourteen thousand of the faithful had fallen victims to putridity in their blood vessels, in consequence of malaria having gained admission to their blood. Having closed these pious labors in the character of "*Old Mortality*" on the Euphrates, he accompanies Bruce into Africa, to do the same kind offices to those, who, had died of putrefaction, under the septic influence of the miasms of the Nile. Having finished his work in the land of the Ptolemies, he vaults, at a single rebound, over the Mediterranean and the Atlantic, and lights unharmed in Duchess county, in the State of New York. And what does he find there, to edify his class first, and the world afterwards? He finds *four personal friends*, two of whom were *wine* and the other two *water-bibbers*. And, while the latter suffered from misasmatic affections, in the autumn, the former escaped. From this is drawn the sweeping conclusion, that *wine is a preventive of autumnal fever!* and water the reverse! a terrible rebuke to our *tetotal* temperance-mongers!

That this may be true in some instances, nobody disputes. But is it *generally* true? Though we shall not answer decidedly in *the negative;* a *positive* answer is still less to our taste. This is one out of many cases, that present themselves to medical writers, and perhaps more especially to medical teachers, in which no decided opinion should be given—in which indeed no such opinion can be given, without doing mischief. To be serious.

Was Professor Hosack certain that the only difference in the habits and modes of life of his Duchess friends, was that of mere wine and water drinking? Did there exist no difference in their diet, as regarded quantity or quality, mode of preparing it or the time of eating it? and were the individuals exactly alike in their habits of clothing and exercise, and in the kinds and degrees of their exposure, as well to the remote, as to the exciting causes of autumnal fever? Were they again of the same age, the same temperament, and the same general character of constitution? Were they acclimated alike to the condition of the atmosphere of Duchess county? Or were some of them older residents of the place than the others? These and several other points should have been gravely considered by Professor Hosack, and due weight allowed them, in maturing his judgment, as to the reason why his wine-drinking friends escaped malarious fever, while the water-drinkers suffered from it. But his work, from beginning to end, shows him to have been much more of a stern uncompromising dogmatiser, than of a calm, substantial, and profound reasoner. Of the latter characteristics he possessed but very little; of the former, by far too much for a standard teacher or writer in medicine. Where he applied once for a decision to the authority of nature and reason, he applied scores of times to the authority of the opinions of men. Hence, as respected himself, his own notion, no matter by what process it was formed, or from what source derived, constituted his only high-court of error; from whose decision he willingly admitted of no appeal. There was, in relation to these matters, a singular anomaly, we might call it *contradictoriness*, in his character. While he referred to the authority of all great men, whose opinions he could learn, he rarely concurred in them, and received with no toleration the slightest difference from his own opinion.

But we are not yet done with our author's almost boundless peregrination, in pursuit of those two arch-enemies of human health and happiness, "miasma and cantagion." Accordingly we are again to accompany him to the banks of the Tagus, where he joins issue with the "remittents" of Portugal, and then to the shores of the Ganges and the Boorampooter, where we find him in close and determined grapple with the "jungle-fever." His next plunge (and it is a bold one) is into the low-lands of North Carolina, where he learns from Dr. Hugh Williamson, who was better informed in almost every other branch of knowledge, than in that of medicine, that even the "most simple *species* of intermittent is in some measure the effect of a *putrefactive* taint introduced into the system." We were not apprized, until we had read this clause by our author, that "intermittents" were held to be divisible into *different* "species." We had been taught to believe that all the forms of intermitting fever belong to the *same* "*species*." Such looseness of language in a public teacher, whether of medicine or of any thing else, is quite unfavorable. It is indicative moreover of a *looseness of*

*thought.* An accurate thinker is equally accurate in his choice of words. And an accurate teacher will make accurate pupils—and the contrary.

The Professor now takes a most devious and anachronous ramble, touching at different parts, and seemingly in different periods, of the world, to collect the opinions of the following individuals on the subjects of miasma and febrile contagion, referring to the names in the same order in which we proceed to record them.

"Sir J. Pringle, Dr. Brocklesby, Dr. Donald Monro, Dr. Wilson, Baglivi, Dr. Rollo, Dr. Melville, Dr. Moseley, Dr. Lind, Livy the historian, Dr. Francis, Dr. Davis, Dr. Willan, Dr. Gregory, Dr. Bateman, Dr. Beddoes, Dr. Price, Dr. Priestley, Murat, Simond, Dr. Cleghorn, Dr. Jackson, Mr. Bartlett, Dr. Thompson, Raymond, Badenock, Bontius, Lysons, Clarke, Mr. Stevens, Dr. Hamilton, Dr. Bayley, Dr. Rush, and Dr. Chisholm—and perhaps others whose names I may have overlooked.

Such is the ponderous and disjointed cluster of authorities, references to which the Professor unmercifully inflicted on his class, in the course of a single brief lecture, merely for the establishment of a few points in pathological science, most of which no one doubts, and of which no tolerably educated physician is ignorant. Some of these points, to which birth is given after such mountain-labor, are as follows:

In our own and other similar climates, miasma exists in greatest abundance in the autumn. Then are enumerated a few circumstances favorable to the operation of miasma on the human system. These are—

*First*—moisture and depressed situations; *second*—woody places. That the influence of this circumstance is ambiguous, might be easily shown. But we are now stating our author's opinions, not our own. *Third*— the cold of night air (*ay, and its moisture, too*) and all great and sudden vicissitudes of atmospheric temperature ; *fourth*—the mixture of sea-water with the water of marshes. This position being laid down, our author, either to display his *ambidexterity* in argument, or, in the belief that "second thoughts are best," (we know not which,) steps forth as the champion of the opposite opinion, and, by *counter-authorities*, (every thing is done by him through the same channel,) he invalidates, in his own mind, his first authority. Why does he believe it invalidated? Because it is sustained by only *one* name ; while the opposite opinion is backed by *two*. Sir John Pringle alone contends that a mixture of salt and fresh water renders a marsh sickly, whereas Dr. Jackson and Mr. Stevens deny it, and espouse the contrary belief. And, with our author, whose opinion seems to have been here, that the majority must rule in science as well as in politics, this turned the scale, and established his dogma.

As respects the fact, there is no doubt that salt-marshes, daily overflown by sea-water, are much less insalubrious than those which are the product of fresh water. For this, several reasons might be rendered ; but we shall content ourselves with *one*. The daily sweep of the tide not only prevents putrefaction from rising to a high pitch ; it also absorbs and carries off the chief part of the malaria that may be produced. That the odour of salt-water marshes is strong and exceedingly unpleasant, is true ; but the odorous gas does not appear to be a febrile poison.

Professor Hosack's view, as to the mode of production of malarious fevers, and their nature when produced, are contained, and show themselves with sufficient clearness, in the following clause:

"Miasma, no doubt, operates as a *ferment upon the whole system*, having its peculiar laws, like every other poison introduced."

Luminous, profound and comprehensive as this sentence was probably thought by the Professor himself, as well as by his friends and admirers, we assert that it does not contain a single definite and intelligible idea. What is the "*ferment*" alluded to? A mere *notion*, as empty and undefined as was ever uttered. What, again, are its "peculiar laws," and what the "laws of other poisons" to which they are likened? Who will answer these natural and necessary questions? Not one. Why? Because they are unanswerable, on account of what they relate to being *nonsensical* and *absurd*. The whole process of *notion-making* here practised, is one of the veriest *soap-bubbles* of the mind that has ever been set afloat. It consists in an unmeaning attempt to illustrate a thing not understood, by comparing it to another thing no better understood. In other words, it is an effort to bring *something* out of *nothing!*

Nor are we able to think much more highly of the Professor's mode of treating malarious fever, especially if applied to those that prevail in the Mississippi Valley. His views on this subject may be sufficiently understood from the following singular and expressive sentence:

"Colonel Howell's family, too, in New Jersey, was attacked with fever, in consequence of cutting down a wood that separated them from a *morass* in the neighborhood. Before that operation, they had been healthy; but the consequence of this change was, that most of the family were attacked with fevers—three died—eight or ten recovered by means of *blisters* and the *free use of bark*, *wine* and *snake-root!* Similar facts have frequently occurred in the West Indies."

Suppose a case of the kind to occur on any of the large streams of our valley, (that referred to by Professor Hosack was near to the Delaware.) A family, we say, consisting of a dozen or more of souls, are attacked with autumnal fever on the Ohio or the Wabash, and, instead of bleeding, purgatives, and antimonials, they are freely blistered, and copiously drenched with barks and wine—suppose such a proceeding, we say, what proportion of said family will recover? Without attempting a specific answer to this question, we say distinctly, that, if *one recover*, he will do so in *spite* of the doctor and his ministry! The practice would be murderous. Yet are we assured, by the most distinguished of the Philadelphia Professors, that the "*practical part* [of the Lectures we are examining] *he considers sound, or at least that it corresponds very much with his own views.*"

To "LECTURE XV.,"

our author gives, as its heading, the sufficiently significant terms of

"Contagion and its Laws."

To follow the Professor through the whole extent and doublings of his long, and labored, and tortuous homilies on this subject, is not our intention. We have neither time nor patience to execute the task; nor would we deliberately inflict on our readers the severe trial of reading the result of such a disquisition. The toil of the analysis, moreover, is unnecessary, in as much as all our author's views on the subject have been long since and repeatedly examined and refuted. As respects the contagious nature of yellow fever, in favor of which the Lectures before us partinaciously contend, that, as far as our information extends, is now believed in by no one who has spent even a day in seriously examining it, under favorable circumstances. Except with a few, who have never seen a case of yellow fever, and are ignorant of it, of course, as well in its nature as in all its relations, a belief in the contagious quality of that complaint, is as obsolete, and deemed as much of a superstition, as a belief in witchcraft. As respects ourselves, we assert, without hesitation, that we can adduce as much *solid*, or even *highly plausible*, matter in support of a belief in the contagiousness of rheumatism or intermitting fever, or even of mental insanity, as any one can in support of the notion of yellow fever contagiousness. To descant on that topic, therefore, we should deem an unpardonable waste of time. To a very brief discussion, then, of the *supposed* contagious quality of *oriental plague* alone shall we devote a few pages.

In no respect essential to the constitution of the most striking *similarity*, or even of *virtual identity*, does that complaint differ from yellow fever. The points in which the two diseases differ most from each other, are neither *radical* nor *indispensable* to them. A jaundiced skin and black vomit, though frequent accompaniments, make no *necessary* part of yellow fever; and plague is not always free from them. And buboes and carbuncles, though most frequent in plague, are not essential to it, and have been seen also in yellow fever. With the exception of those four affections, three of which are *local externals*, and the fourth a *non-essential*, the complaints are the same, only modified by the peculiarities of different countries, and of the constitutions of their inhabitants. They begin under like circumstances of time and place, attack most readily persons of the same age and description, the course and duration of individual cases are alike, they spread under similar circumstances, and with equal rapidity, and they terminate under circumstances equally similar. Such is the general mass of facts; and if apparent exceptions occasionally occur, that is no more than occurs in all other cases, and they may be easily explained.

Plague and yellow fever terminate their career at the commencement of cool weather, when malaria, the result of putrefaction, ceases to be generated, and when that already in existence is precipitated or otherwise holds no longer possession of the atmosphere. After this, there is no further dread or suspicion of contagion, until the next return of hot weather—for both complaints are hot-weather productions. Immediately on the close of those epidemics in the autumn or winter, houses depopulated by them are entered, *without purification*, by fresh families, beds where the sick had lain and died are slept on *unwashed*, the furniture found in the dwellings is familiarly handled and used, and all this is often done under a great want of cleanliness ; and yet no one sickens in consequence of it from plague or yellow fever, until the return of another malarious period ; and then no caution nor cleanliness is a safe-guard to health. To persons well acquainted with the history of plague and yellow fever, these facts are as familiar as the vicissitudes of the seasons. Nor is this all.

In a pure and unvitiated atmosphere the two diseases are alike incommunicable, and alike unsuspected of the qualities necessary to render them communicable. That yellow fever is never communicated from the sick to the well, on the heights of the Schuylkill, remote from the pestilential atmosphere of Philadelphia when visited by the malady, nor on the heights around New York and Baltimore, under similar circumstances, is a fact as thoroughly established, and as confidently believed, as any other in physical science.

Of the oriental plague, the same may be affirmed. On the heights round Constantinople, Smyrna, and Alexandria, it is perfectly well known to be an *incommunicable* complaint.

Nor will any one versed in its history deny this assertion. By Dr. Russell himself, the great apostle of contagion, and by every other writer who has adverted to the fact, its truth is acknowledged. The cause of this is plain. On the heights referred to, the atmosphere is free from a pestilential taint; and, by the bodies of the *sick*, no contagious matter is secreted to poison the *well*.

But, say our author and his adherents, plague *is* contagious; because it has been communicated, by inoculation, from the sick to the well. No such thing, we reply—no such thing! Plague has never been thus communicated, any more than common bilious fever. Nor, as far as we are informed, has the experiment ever been properly performed.

True, into the legs or arms, hands or shoulders, of individuals who had been *previously exposed to a pestilential atmosphere*, matter from buboes or carbuncles, or both, has been inserted; and the persons have been afterwards attacked by pestilence. And so would they have been, just as readily, had they not been inoculated. Why? Because they had derived the poison of the disease from an unsound atmosphere.

But in a pure and healthful atmosphere, remote from the sphere of pestilence, inoculate, with the so called *pestilential fluid*, a person who has never been within that sphere, and let him not approach it afterwards; and the inoculation will be fruitless. The health of the individual will remain unimpaired; except perhaps some local and temporary irritation which may be produced by the insertion of the vitiated fluid into his flesh, as happens not unfrequently to the dissection of dead bodies, when they scratch or puncture their fingers with their scalpels. Is the specific disease, of which the person under dissection died, thus communicated to the dissectors? No; nor will plague be communicated by inoculation with the vitiated matter from the body of a patient laboring under that malady. We fearlessly hazard our opinion, and reputation, that, by a thousand experiments thus cautiously and skilfully performed, not a single attack of plague will be produced. In a word; as soon as the notion of the contagious nature of plague shall have been scrutinized as severely and judiciously, as has been that of the contagiousness of yellow fever, it will be with equal certainty dissipated and consigned to the class of *untenables* and *obsoletes.* And thus shall we be free from the trammels and immense evils—of one more of the superstitions that have come down on us from ages of ignorance and error.

To give the work before us another opportunity of *speaking* for itself, and our readers, also another of *judging* for themselves respecting its merit, we shall extract and publish entire, "Lecture LVII, on Rheumatismus vel Myitis." This lecture constitutes perhaps as fair an average, in matter and manner, of what the book contains, as any other we could select. We beg our readers to peruse it carefully; and if they can derive any benefit from a farrago, which to us appears indefinite, pointless, and in many points all but *meaningless,* we wish them joy of it. And might we venture to tender respectfully, a word of advice to the Reverend Editor of the volume, it would be, that he confine his professional labors hereafter to his own vocation, in which we are told that he is not a little distinguished.

### LECTURE LVII.

### RHEUMATISMUS VEL MYITIS.

Rheumatism, the myitis of Crichton, thereby denoting the seat of this disease to be chiefly in the muscles. By Dr. Parr the old name of arthritis is retained, which by the ancients was applied both to gout and rheumatism. It is important that they should be separated, both as regards their nature and their treatment. With this view too Dr. Parr annexes to the term arthritis, the adjunct rheumatismus ; gout he calls arthritis podagra. I have retained the term rheumatism, as pretty generally agreed upon among writers. It is so called from rheuma, (from the verb *rheo*, fluo,) denoting a defluxion on the part affected ; and answers the purpose as well as any other name proposed ; and has this advantage, that it is well understood. Dr. Cullen's definition contains many details, viz. " morbus ab externa, et plerumque evidente causa ; pyrexia ; dolor circa articulos, musculorum tractum sequens ; genua, et reliquos majores, potius quam pedum vel manuum, articulos, infestans, calore externo auctus." He intends by the first part of this definition to distinguish it from gout ; but this is not the place for diagnosis. A definition should contain only the characteristic symptoms of a disease. The first part of his definition is not invariably true, that it proceeds, (in all cases,) from an external cause ; nor is the last uniformly true, that it is increased by external heat. Besides, these circumstances are equally true of any of the phlegmasiæ ; they are applicable to gout as well as to rheumatism, for gout frequently, like rheumatism, is excited by cold ; and, like rheumatism, is increased by heat. Rheumatism, acute, Dr. Home observes, is more frequent in summer than winter ; attacks more females than males ; that it is rather a nervous than inflammatory disease ; that antispasmodics are most effectual in curing it, but, by the by, venesection is the best antispasmodic ; that of twenty-two patients in the infirmary with it, sixteen were females ; that he has more in summer than winter ; but his observations are made at the infirmary. Query : Are there not some circumstances probably overlooked, which will otherwise account for these occurrences, so contrary to general observation ? That it is not the heat of the bed which aggravates the disease more at night, but what he calls a nocturnal paroxysm, observing that the patients with it are always in dread. This same remark will extend to most inflammatory diseases.

Another exception to Dr. Cullen's definition is, that it is not confined to the joints : on the contrary it frequently affects the muscles of the head, of the chest, of the abdomen, and even the viscera, as well as the joints ; and it affects the small joints as well as the larger. I know a lady, afflicted with a rheumatism, who had large deposits of chalky matter, similar to those of gout. Dr. Gregory has also frequently made this observation, that rheumatism affects the small as well as the larger joints; that he has seen the fingers crooked with it, and then of course incurable. Dr. Cullen, therefore, should have added the qualifying expression, "plerumque," after "dolor." Indeed, rheumatism might be defined to be an inflammatory disease of the muscles, (including their fasciæ, or inclosing membranes,) and the membranes composing and surrounding the joints. And like the inflammation of some of the viscera, it might also admit of the distinction into membranous and parenchymatous, showing by the former, that the disease is more immediately seated in the membranous portions and coverings of the muscles, and of the joints; and that the latter more especially affects the strictly muscular and cellular portions of the muscles; and in which the pain and febrile symptoms are relatively less acute, which sometimes terminates in abscess. In the definition in my nosology, I have accordingly adverted to these particulars. Read there my definition of rheumatism. In my definition of species, I have complied with custom in dividing it into acute and chronic. Yet I wish it to be understood that I consider chronic rheumatism, as chiefly the sequela of the former; a morbid sensibility that remains in the part after the acute or febrile rheumatism has run through its course, and the evidences of general excitement have subsided, as scirrhus succeeds to inflammation. Accordingly Dr. Cullen places chronic rheumatism under a distinct head of arthrodynia or pain of the joints; and considers it, very properly, among the consequences or terminations of rheumatism; and which he thus defines: "Post rheumatismus, nisum violentum, vel subluxationem; dolores artuum vel musculorum, sub motu præsertim, aucti plus minusve fugaces, calore lecti vel alio externo levati; artus debiles, rigidi, facile, et sæpe sponte frigescenter; pyrexia nulla; tumor plerumque nullus."

Some allege that rheumatism sometimes takes place from the presence of an inordinate quantity of lithic matter in the system. The phenomena arising from this supposed cause, are more satisfactorily to be explained by the interrupted state of the excretions, and the febricula such interruption produces, attended with pain, and other irritations of the debilitated sensitive system; for these occur most usually in advanced life. And from the same causes, the impaired state of the excretions, we may account for the inordinate deposit of earthy matter in different parts of the body.

The symptoms of accute rheumatism are those of the phlegmasiæ in general. It usually comes on with chills, succeeded by great heat, with pain in the limbs, changing its seat from one joint to another, and is especially increased by the heat of the bed, and occasionally attended with great soreness to the touch of the surface as well as of the internal parts. The pulse is hard and frequent; the tongue white and furred, with great thirst. The secretions are generally interrupted: the urine appears pale; afterwards becomes high caloured, depositing a large lateritious sediment, as in gout; but the patient still is not relieved. Indeed we shall find that gout is rheumatism in the small joints; and rheumatism is gout in the muscles and larger joints, though usually proceeding from different causes, and occurring at different periods of life. Stoll mentions that they are only varieties of the same disease. (Rat. Med. Part 3, p. 137, 430.) And Bergius believes that they are convertible diseases. The fever continued, and a direction of the fluids taking place to the limb, or parts affected, swelling ensues, oftentimes resembling that which is attendant upon the cruritis or phlegmasia dolens of lying-in women; and, like that, affording the patient some release from his sufferings; for the pain is frequently suspended by the pressure upon the nerves of the limb. This disease occurs at those seasons of the year which are most changeable, as the spring and autumn, not so much so in the uniform and steady cold of winter, nor in the warm seasons of the year, or in warm climates, with the exception of bathing in cold water when heated, or lying on the damp ground when the body is perhaps under excitement. But in the country it is relatively a disease of frequent occurrence, owing to the moisture of the earth, and of the atmosphere, to which the inhabitant of the country is exposed.

Its terminations are, by resolution. Sometimes metastasis, as to the head, the lungs, and the heart, especially where the practice is feeble. (See Bedingfield, p. 307.) Abscess sometimes. More commonly in a gelatinous affusion; and in deposits of earthy matter. In this it differs from many, nay most of the phlegmasiæ, except it be the gout, to which it is most nearly allied. When it ends by resolution, I have known a case where it assumed the intermittent character, the pain returning at a regular hour upon alternate days, but with very little fever. This is called by Alibert, the rheumatic form of intermittent.

### DIAGNOSIS.

It is distinguished from gout by the cause and time of life. The ancients knew nothing of rheumatism, as distinct from gout. Sydenham was the first who made the discrimination. He had experienced gout himself, and saw rheumatism in others. Boerhaave too had rheumatism eight months. The best account of it is in Van Swieten's Com., a book shamefully overlooked.

Gout usually appears after thirty-five ; rheumatism before that period of life ; sometimes at five or six years of age. Gout most usually arises from the sanguine and plethoric habit of body and free living.

Rheumatism from cold. In rheumatism, too, the fever is continued. In gout, most commonly, it intermits ; at least a sensible remission is apparent. In gout the stomach is principally affected ; not so in rheumatism.

Nephritis is distinguished from it by the peculiar symptoms that attend upon the organ diseased, as connected with the stomach, the testes, and the round ligaments. Boerhaave, strange to tell, in his own case, committed this error by confounding nephritis and lumbago ! ! Bending the body alone, in nephritis, should decide it ; whereas in lumbago the body cannot be bent without great suffering.

Hydrargyria is another disease, produced by mercury, that is very similar in its symptoms, but differs only in its cause. Indeed in the relaxed state of the exciting and sensitive condition of the nerves, it is not improbable that cold, in most cases, produces it, even though mercury be acting on the system.

" A course of mercurial medicines," says Heberden, " has, with great reason, been suspected of bringing on something like this distemper (chronic rheumatism) in many persons ; and it has appeared to do so in the same person five or six times ; i. e. as often as the mercury was repeated." (Heberden, p. 501.)

Arsenic too produces rheumatism, whether directly or by adding to the irritability of the system, is not so easily decided. In those countries where it is much employed in the treatment of fevers, rheumatism is of frequent occurrence.

### PREDISPOSING CAUSES.

1. Fulness of habit by creating an inflammatory state of the system. Sir Clifton Wintringham remarks, that those who have undergone amputation of a limb are thereby predisposed to this and other inflammatory diseases. G. M. lost a limb. He afterwards became plethoric ; gout followed ; and from this disease he suffered severely, notwithstanding the anticipations of the person who congratulated him upon the cost of his limb, believing that God had great things in store for him, by taking away one of his legs. Upon this occasion, he addressed Mr. M. very earnestly. Mr. M. replied, "Sir, you really speak so eloquently upon this subject, and hold out so many blessings for me, in consequence of the loss of my one leg, I am almost induced to lose the other also." (Comm. de Morb. Quibusdam, Art. 79.) But to return: 2. A sanguine temperament. 3. Vigour of early life; i. e. from childhood to thirty-five. The young, the vigorous, and the active. Hence, too, we infer its inflammatory character, independently of its symptoms. 4. Intemperance will predispose to it. 5. A former attack, by which the parts affected are rendered very sensible to the exciting cause.

### EXCITING CAUSES.

Cold, heat, or the two alternately applied; violent exercise; change of dress; intemperance; wet clothes; cold bathing when heated.

### TREATMENT.

Venesection, general, repeated three or four times. As Dr. Gregory used to remark, there is very little danger, as it regards the patient's life, but he is in great danger of losing the use of his limbs if you do not industriously cut off the flow of blood to the inflamed part. Gregory's bleedings were too small; viz. about ℥xij.; hence he found it necessary to repeat them. Local, by leeches; cupping repeated, not regarding the buffy coat, for the buffy coat exists in the last stage as well as the first. Thomas remarks, that in rheumatism the buff increases as the disease advances. So also in intermittents. (p. 157.) Dr. Gregory makes the same remarks. Even after one hundred and seventy ounces have been drawn, the disease has, in some cases, been unsubdued. This treatment was therefore condemned as erroneous by Dr. John Fothergill, and by Dr. Haygarth, who then prescribed the bark! Fordyce, too, came into their views. (See Thomas, p. 186. See also Clin. Hist. of Diseases, Edin. Journal, Vol. 1, p. 482.)

Saline purges—A course of James' powders. Sudorifics—Warm bath; fomentations; sinapisms, moxa. Blisters—Ointment of tartarized antimony. Liniments—volatile; camphor, dissolved in oil, or the volatile liniment, preferred by Dr. Good to the spirituous liniment, which he supposes to dry the skin too much; which heats and stimulates without exciting moisture; a waistcoat of coarse, brown sheething-paper produces a diaphoresis, and excites by the tar with which it is so largely impregnated; oiled silk. Diuretics—Nitrate of potash. Dr. Brocklesby's great remedy was nitre. Dr. Kuhn's also. It was combined with the tartrite of antimony. Digitalis has also been by some recommended on account of its diuretic properties.

Anodynes are very proper after the inflammatory state has been subdued by depletion; in that case, Dover's powders may be very beneficially prescribed. It was in this disease that Dr. Dover first introduced this valuable combination of opium, ipecacuanha and the sulph. of potash. He became celebrated in early life as a captain of one of the privateers in Queen Anne's time, that sailed round the world. In the last of his life he distinguished himself by the introduction of this powder; and by another practice he introduced, of giving crude quicksilver—he took it himself in large quantities, and became so attached to it that he said, if he could afford it, he would swallow a pound of quicksilver daily—the facetious Dr. Dover, as Fuller calls him. Garthshore and Sir John Pringle as well as most practical writers, concur in the approval of this sudorific anodyne, which preserves a pervious state of the skin, at the same that it allays the sufferings of the patient. It should be renewed every three or four hours, until sweating is produced—renew it in twelve or twenty-four hours to continue the sweat. Rhododendron chrysanthemum of Linnæus, also once much in use, both in gout and rheumatism—a native of the snowy Alps and mountains of Siberia—in Russia also much employed, says Dr. Guthrie. (Med. Comm. vol. 5, p. 434.) Three or four doses generally giving relief. (See also Pearson's Mat. Med. Home's Clin. Exp.) Another remedy that has come into use in acute rheumatism, is the Peruvian bark. Dr. Haygarth, Dr. Fordyce, and Dr. Duncan, as well as others, viz: Sir Geo. Baker, Dr. Heberden, Dr. Saunders, Dr. Willen, Sir Lucas Pepys, Dr. Lettsom, Dr. Aikin, and Granger, are the advocates of this practice. (See Med. and Surg. Remarks.) Dr. Haygarth introduced it as early as 1772. In the hands of others it has not been prescribed with equal success—on the contrary, with decidedly injurious consequences. Dr. Cullen had reason to think it injurious. Dr. Haygarth was so convinced of its superior efficacy, that he thought the bark did not cure an ague so quickly and so successfully as it does acute rheumatism!! Dr. Gregory disapproves of it in the first stage—in the greater proportion of the cases it did no good. He observes, it is well to keep it in view, especially in cases where you fail to effect a diaphoresis; this last is questionable practice. Bedingfield observes that it is generally injurious in rheumatism. Thomas uses it but in combination with nitre. I have seen it prescribed by Dr. Duncan, in the Infirmary of Edinburgh, in several cases, with the most favourable results, while in others, with an aggravation of the disease, altogether depending on the stage of the disease and the habit of body in which it was administered. It is the want of attention to these particularly, and the want of discrimination on the part of the prescriber, that has led to these different and opposite results that have been noticed by practical writers. When the disease is attended with the symptoms of active inflammation and general excitement, the bark as well as other stimuli, must be injurious. But there are cases wherein local inflammatory symptoms may be continued after the general excitement is taken off, that is, partial excitement continues. The effect of increased sensibility of the part, attended with a small pulse, cool or cold extremities, and the general powers of the system greatly impaired and reduced by the depletion the patient has undergone. Thus we often see a headache, a sore throat, an inflamed eye, or other local affection, are sometimes thus continued, that is, by the stimulus of relaxation, as Mr. Hunter calls it; in other words, irritability or susceptibility, which is only relieved by tonics and stimulants that shall distribute the excitement throughout the system, and which at the same time shall diminish the sensibility of the part affected; so of rheumatism. There are cases where stimuli are especially useful in this way; and in like manner, there are cases where the sensibility of frame is such that even the whole arterial system shall partake of it, and a degree of fever be produced that as in typhus, hectic, scarlatina, and other fevers, is only to be counteracted by stimuli and tonics. In such cases the bark may diminish both pain and fever, by imparting tone to the system, and thereby lessening sensibility which is frequently the accompaniment of weakness. In cases of this nature, however, much discernment and practical observation are required to know when the depletion is to be persisted in or tonics and stimuli are to be administered.

The diet should be the strictest abstinence during the synochal stage of rheumatism. No animal food in any shape—on the contrary, a cool, spare diet; milk whey, buttermilk, ripe fruits, gruel panada, rice, &c. I have known the obstinacy of patients and their friends, in this disease, by the use of animal food, to render all that had been done of no avail, by the excitement being renewed by the use of animal food. Diluents—mineral waters. Regimen—do not load the patient with great quantities of clothing, or bedding. Flannel or cotton next the skin are assuredly necessary and proper ; but great care to avoid abuse as to their quantity.

We now proceed to notice that form of rheumatism which is called chronic, the arthrodynia of Dr. Cullen. For the most part, as already observed, this is usually the consequence of preceding attacks of the acute or inflammatory rheumatism ; and is continued as the effect of great sensibility of the part, and of the whole system. In some cases the secretions are checked, accompanied with a slight degree of fever. Aged persons thus are frequently affected by pain connected with some febrile symptoms induced by a constriction of the surface, or perhaps by debility in the extreme vessels. Not unfrequently, too, they are the effect of local injuries, as violent exertions, falls, strains, or bruises of the muscles or joints, to which the aged are particularly exposed. In those cases, mild evacuations by the bowels and the use of some diaphoretics will be at first proper; but as soon as fever is altogether removed, as in the last stage of acute rheumatism, stimuli are called for, especially such as are calculated to preserve an open state of the surface of the body. The Peruvian bark is had recourse to by some, bitters by others; chalybeates, in the form of chalybeate waters, or iron in substance; given in the tinct. volatile ʒii. ter in die in milk, decoction of the woods, with camphor. ℞. guiac ʒvj., camphor ʒi., opii. ʒij., tart. emet. ʒi. M. divide in pill 120—two to be given three times a day. Turpentine in form of the oil ʒi.—℥i. of honey, a teaspoonful occasionally. Local stimulants—plasters, the emp. calidum, pix burgund., tart. emet. ointment, the Hungarian plaster. Liniments—soap lin. with aq. ammon. aa. ℥ij., turpentine, essence of mustard, stramonium ointment, spirituous baths, hot baths—106° Fahrenheit—friction with flesh brush, flannel. This was constantly the practice of the Greeks and Romans; the Chinese, too, in health, to prevent disease. The Emperor Augustus, it is said, was so completely curried that his skin exhibited the effects of the instrument. Flannel shirts,* bandages, rollers, knee cap. (See Balfour on bandages in rheumatism. Med. Repertory, vol. 6. p. 19.) A slight mercurial action in the system is recommended by Bedingfield, by giving calomel ℈i. twice a week. The dracontium fœtidum, or the skunk cabbage, is advised by Dr. Thatcher, in his medical practice ; of the dried root he administers xx. or xxx. grains, three or four times a

* A flannel shirt, Dr. Gregory used to tell us, was worth half a dozen of any other remedies in this disease.

day, or an infusion of it. Phytolacca decandra, or poke weed, has been recommended, in the form of an extract from the leaves, or of a tincture of the leaves. Tinct. colchicum is also advised.

The diet should be generous, such as wine-whey, porter, animal food. Condiments, as mustard-seed, horse-radish, cayenne, &c.

REGIMEN.

Let the dress be flannel, frequently changed; exercise, especially a journey. If confined to a chamber, the dumb bell, that is, a mass of metal with a rope fastened to it and passing it over a pulley, pulling it up and down, as in ringing a bell.

If we have been hitherto silent in relation to the style of these "Lectures," it is not because we regard the style of scientific and professional writings as a matter of indifference. Far otherwise. Though we should condemn the overloading of such works with labored and powerful decorations of expression, we have yet to be informed of any good reason, why they should not be written as purely and chastely, as correctly and perspicuously, as other forms of composition. Why should matters of history, poetry, and moral fiction be presented to the public eye, decked out in all the beauties and decorations of language and imagery; and the still more important matters of science and profession be brought forward, covered with apparel that can scarcely be called decent? We are not unacquainted with the reasons assigned for this difference; but to us they are unsatisfactory. We think that science not only deserves, but demands a style both sound and clear, classical and vigorous.

Without pronouncing the style of the "Lectures" peculiarly faulty, we are warranted in saying that it is equally remote from being unusually creditable. Though we do not call it either vulgar or obscure; it is coarse, crude, and far from being definite. The words employed in it are neither tastefully selected, nor judiciously collocated. The writer would seem to have been too much engrossed by the language of the Greeks and Romans, to have bestowed the requisite attention on his own—much more anxious to be accounted a profound Greek and Latin, than a well-disciplined Belles Letters scholar. And we regret to say, that this is too generally the case, with all vain-glorious votaries of the dead languages. And it is all wrong, instead of a mere smattering of Greek and Latin (and none of us acquires, or can acquire more,) the true glory of American scholarship consists in a thorough scientific knowledge, and practical command, of the unequalled resources of the Anglo-Saxon tongue. C. C.

